# Preparing the Gut with Antibiotics Enhances Gut Microbiota Reprogramming Efficiency by Promoting Xenomicrobiota Colonization

**DOI:** 10.3389/fmicb.2017.01208

**Published:** 2017-06-28

**Authors:** Shou K. Ji, Hui Yan, Tao Jiang, Chun Y. Guo, Jing J. Liu, Shuang Z. Dong, Kai L. Yang, Ya J. Wang, Zhi J. Cao, Sheng L. Li

**Affiliations:** ^1^State Key Laboratory of Animal Nutrition, Beijing Engineering Technology Research Center of Raw Milk Quality and Safety Control, College of Animal Science and Technology, China Agricultural UniversityBeijing, China; ^2^College of Animal Science, Tarim UniversityAlar, China; ^3^Jinzhong Vocational and Technical CollegeJinzhong, China; ^4^College of Animal Science and Technology, Shihezi UniversityShihezi, China; ^5^College of Animal Science, Xinjiang Agricultural UniversityUrumqi, China

**Keywords:** fecal microbiota transplantation, colonization efficiency, gut pretreatment, antibiotics, microbiota reprogramming

## Abstract

Gut microbiota plays multiple important roles in intestinal and physiological homeostasis, and using fecal microbiota transplantation (FMT) to reprogram gut microbiota has demonstrated promise for redressing intestinal and physiological disorders. This study tested the alterations in reprogramming efficiency caused by different gut preparation procedures and explored the associated underlying mechanisms. We prepared the guts of mice for FMT by administering one of the three most-clinically used pretreatments [antibiotics, bowel cleansing (BC) solution, or no pretreatment], and we found that preparing the gut with antibiotics induced a more efficient modification of the gut bacterial community than was induced by either of the other two pretreatment types. The increased efficiency of antibiotic treatment appeared to occur via increasing the xenomicrobiota colonization. Further analysis demonstrated that antibiotic treatment of mice induced intestinal microbiota disruption, mostly by expelling antibiotic-sensitive bacteria, while the indigenous microbiota was maintained after treatment with a BC solution or in the absence of pretreatment. The amount of antibiotic-resistant bacteria increased shortly after antibiotics usage but subsequently decreased after FMT administration. Together, these results suggest that FMT relied on the available niches in the intestinal mucosa and that preparing the gut with antibiotics facilitated xenomicrobiota colonization in the intestinal mucosa, which thus enhanced the overall gut microbiota reprogramming efficiency.

## Introduction

The intestinal microbial ecosystem plays a variety of important roles in animal physiology and gut homeostasis (Clemente et al., 2012; Tremaroli and Bäckhed, 2012; Boulangé et al., 2016). Intestinal microbial disorders have been demonstrated to be related to multiple human diseases such as *Clostridium difficile* infection (CDI; Fuentes et al., 2014; Bagdasarian et al., 2015), inflammatory bowel disease (IBD; Marchesi et al., 2016; Ishikawa et al., 2017), obesity (Ridaura et al., 2013; Boulangé et al., 2016), and mental illness (Foster and McVey Neufeld, 2013; Zheng et al., 2016). Consequently, reprogramming gut microbiota is a promising approach to redressing intestinal and physiological disorders (Cammarota et al., 2014; Petrof and Khoruts, 2014; Zheng et al., 2016).

Fecal microbiota transplantation (FMT) is considered an efficient approach to gut microbiota reprogramming through introducing xenomicrobiota into the gut (Cammarota et al., 2014; Khoruts and Sadowsky, 2016; Li et al., 2016; Manges et al., 2016), and has been recommended to be performed therapeutically (Bagdasarian et al., 2015; Cammarota et al., 2017). The reprogramming efficiency of FMT depends on many factors, such as the source of microbiota, methods of microbiota preparation, and administration (Seedorf et al., 2014; Kelly et al., 2015; Vermeire et al., 2016), and especially gut preparation for the procedure (Ishikawa et al., 2017). However, there remains a knowledge gap on how to increase FMT efficiency, and the alteration efficiency of gut microbiota reprogramming by FMT in the gut mucosa has also not been fully elucidated.

Recent data suggest that perturbations in the intestinal microbiota alter host susceptibility to xenobacteria (Sekirov et al., 2008; Mallon et al., 2015), and one potential mechanism for this is niche competition in the mucosa between the xenomicrobiota and indigenous microbiota, based on ecological niche theory (Lee et al., 2013; Caballero et al., 2015; Schluter et al., 2015; Khoruts and Sadowsky, 2016). Based on this, we hypothesized that the reprogramming efficiency could be regulated by perturbing the gut microbiota before FMT administration. Thus, this study had three main objectives: (1) to test whether or not FMT efficiency changed following any of the three most-clinically used gut preparation procedures [antibiotics, bowel cleansing (BC) solution, or no pretreatment], (2) to characterize the influence of gut preparation on the gut microbiota in the mucosa and lumen to gain insight into potential mechanisms, and (3) to monitor the antibiotic-resistant bacteria during period of combination therapy with FMT and antibiotics.

## Materials and Methods

### Mice

Eight-week-old, specific-pathogen free (SPF), male ICR mice were acquired from Beijing HFK Bioscience Co., LTD (Beijing, China). All mice were bred in the Life Sciences animal facility of China Agricultural University in a temperature-controlled (20°C) facility with a 12-h light/dark cycle and received a standard chow diet containing 18% protein, 4% fat, and 5% fiber *ad libitum* with free access to clean water. The feed and water were changed every morning to keep them fresh. Each group of mice was bred in separate cages. All animal studies were approved by the Ethical Committee of the College of Animal Science and Technology of China Agricultural University, and all subjects gave written informed consent in accordance with the Declaration of Helsinki.

### Mouse Treatments

Mice were randomly assigned into three study groups, and each group of mice received treatment (antibiotics, BC solution, or no pretreatment) with one of the most-clinically used procedures before undergoing FMT (Manges et al., 2016). Control mice received no treatment, and all treatment group mice received 500 μl of solution by gavage for 3 days (twice per day at 12-h intervals). The antibiotic pretreatment mice received a cocktail of antibiotics, and the BC pretreatment mice received Moviprep solution. The antibiotic cocktail (AT) was administered at the recommended dose (Morgun et al., 2015) and consisted of ampicillin (8 g), neomycin sulfate (8 g), and vancomycin (4 g) all dissolved in 1 l of distilled deionized water. The Moviprep solution was prepared as described in a previous study (Jalanka et al., 2015); briefly, PEG 3350 (100 g), NaCl (46 mmol), NaSO_4_ (53 mmol), and L-ascorbic acid (30 mmol) were mixed and dissolved in 1 l of distilled deionized water. After sample collection from half of the mice (*n* = 4–5 mice/group), FMT was conducted on the other half of the mice (*n* = 4–5 mice/group).

### Fecal Microbiota Transplantation

Fecal samples from a healthy adult human (based on the subject’s self-report of being without any disease or medicine use in the previous 6 months) were used to colonize the guts of three groups of mice (control group mice received no pretreatment and two treatment groups received a pretreatment with antibiotics or BC) once per day before morning feeding for 3 days. The procedure for preparing the fecal samples for microbiota transplantation was as described in a previous study (Hevia et al., 2015). Briefly, fecal samples were handled under anaerobic conditions, each fecal sample (10 g) was suspended in 50 ml of distilled deionized water, suspensions were extracted and immediately administrated to the recipient mice by oral gavage with 500 μl of solution per mouse. Samples were collected at 7 days after FMT administration finished.

### Sample Collection

Samples were collected before morning feeding. After mice were euthanized by cervical dislocation, the intestinal lumen contents in the middle parts of the jejunum and colon were collected after the intestine was split. The resulting small slices of intestinal tissue (approximately 1 cm in length) were then washed with 10 ml of sterile 0.9% NaCl solution to remove the digesta and non-adherent bacteria, and the intestinal mucosal tissues were collected carefully after washing. All collected samples were placed in cryovials and immediately snap frozen in liquid nitrogen. These samples were transported and stored at -80°C in their original tubes until further processing.

### DNA Isolation and 16S rRNA Amplicon Sequencing

Genomic DNA was extracted using a Qiagen DNA Extraction kit^TM^ (Qiagen, Hilden, Germany) according to the manufacturer’s protocol. Next, 16S rRNA genes were amplified using barcoded primers covering the V3–V4 region. Sequencing libraries were generated using the NEB Next Ultra DNA Sample Preparation kit (NEB, MA, United States) following the standard Illumina sample-preparation protocol (Caporaso et al., 2012) and then sequenced on an Illumina MiSeq platform (San Diego, CA, United States); paired-end reads with ∼420 bp were generated.

### Data Processing

Quality control of the raw data was performed by FastQC (version 0.11.3). Reads with a quality score higher than 30 were retained for further analysis. Paired-end reads from the original DNA fragments were merged using FLASH (version 1.2.7; Magoc and Salzberg, 2011). Paired-end reads were then assigned to each sample according to the unique barcodes. Concatenated sequences were detected using USEARCH (version 6.1) and subsequently filtered out. Sequence analyses were performed using QIIME pipeline (version 1.5.0; Caporaso et al., 2011). Generated sequences were distributed into different samples based on barcodes, and the OTUs were defined by clustering sequences together with a 97% identity cut-off using UCLUST software (version 9.1; Edgar, 2010) after removing the singletons and barcodes. The RDP classifier and Greengenes 13.5 database were used for taxonomic classification of generated OTUs. 16S rRNA gene sequencing reads were deposited in the Genome Sequence Archive^1^ in the BIG Data Center under accession number PRJCA000342.

### Data Analysis

Alpha diversity indices were calculated using QIIME pipeline (version 1.5.0; Caporaso et al., 2011), and the diversity, evenness, and richness were calculated as previously described (Fuentes et al., 2014). The beta diversity indices between samples were determined based on Bray–Curtis metrics with R software (version 3.1.2). A phylogenetic analysis was carried out with ClustalW2 and PhyML3.0 using the maximum likelihood method. PICRUSt and LEfSe (LDA Effect Size) analyses were performed online^2^ to find the different activities of pathways with a *p*-value higher than 0.05 and a LDA score higher than 2. Comparisons between groups were performed using a Wilcoxon test or Kruskal–Wallis test with R software (version 3.1.2). All data are presented as mean ± SD, with ^∗^*p* < 0.05, ^∗∗^*p* < 0.01, ^∗∗∗^*p* < 0.001.

## Results

SPF mice were either left untreated (CON) or prepared for FMT by gavage administration of an AT or a Moviprep solution for BC for 3 days. After sample collection, a FMT with xenomicrobiota from a healthy human was performed, and additional samples were collected to assess the FMT efficiency (**Supplementary Figure S1**). With high throughput sequencing based on the 16S rDNA V3–V4 region, a total of 2,862,280 (49,350 ± 18,901 sequences/sample; mean length: 419 ± 11 bp) and 1,653,597 (57,021 ± 18,065 sequences/sample; mean length: 418 ± 11 bp) high-quality 16S rRNA gene sequences were obtained in the intestinal mucosa after gut pretreatments and after FMT, respectively.

### Alteration of Intestinal Microbiota after FMT with Different Gut Preparation Procedures

**FIGURE 1:**
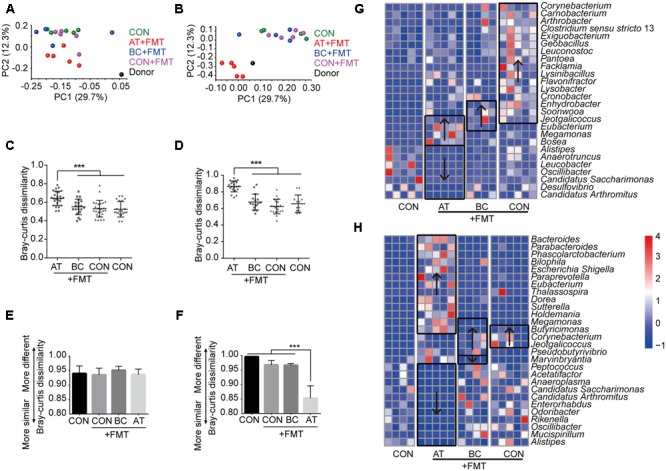
Influence of pretreatment on FMT efficiency. **(A,B)** Principal coordinate analyses of mucosa microbiota plotted on the first two principal components in the jejunum **(A)** and colon **(B)**. **(C,D)** Bray–Curtis dissimilarities using OTU taxa of the jejunum **(C)** and colon **(D)** mucosa bacterial communities after FMT compared with control mice. **(E,F)** Bray–Curtis dissimilarities of the mouse jejunum **(E)** and colon **(F)** mucosa bacterial communities after FMT compared with the donor bacterial community. **(G,H)** The altered taxa in abundance at the genus level in the jejunum **(G)** and colon **(H)**. The significantly altered taxa compared with control mice are enclosed in boxes, and arrows indicate increased (↑) or decreased (↓) abundance. Only taxa with *p* < 0.05 are included. Data are presented as means ± SD. ^∗∗∗^*p* < 0.001. For all figures: FMT, fecal microbiota transplant group; CON, control group; BC, bowel cleansing group; AT, antibiotics group.

The donor and mouse intestinal microbiota were distinct from one another (**Figures 1A,B**), and the various taxonomy levels had different compositions (**Supplementary Figure S2**); therefore, we expected the mouse intestinal microbiota to change after xenomicrobiota were introduced into the gut by FMT. To examine if FMT following different pretreatments altered the intestinal microbiota, we assessed the microbiota Bray–Curtis dissimilarity based on OTUs. The intestinal microbiota of AT-pretreated mice significantly changed in both the jejunum and colon after FMT (*p* < 0.05); however, those of BC-pretreated or CON mice were not altered by FMT (**Figures 1C,D**).

### Influence of Gut Preparation on FMT Efficiency

To determine if the FMT efficiency, which we defined as the dissimilarity between the altered gut microbiota after FMT and the donor microbiota, varied under different pretreatments, we calculated the Bray–Curtis dissimilarities between the jejunum and colon microbiota of mice and those of the donor. None of the pretreatments affected the FMT efficiency in the jejunum (**Figure 1E**), but the FMT efficiency was enhanced after AT pretreatment in the colon (*p* < 0.05; **Figure 1F**). These data demonstrate that FMT efficiency in the colon can be elevated by preparing the gut with antibiotics. However, the colonization pattern in the jejunum may be different from that in the colon.

### Xenomicrobiota Colonization after FMT with Different Gut Preparation

To assess changes in the bacterial community composition after FMT with different pretreatments, we quantified the post-FMT changes in bacterial composition at the genus level with high confidence abundance (>10^-4^ in abundance) (**Supplementary Figure S3**) and analyzed the altered bacterial taxa. In jejunum mucosa after FMT, AT- or BC-pretreated mice each had four taxa that were more abundant, CON mice with FMT had 16 taxa that were more abundant, and AT-pretreated mice had seven taxa that were less abundant compared with controls that did not undergo FMT (*p* < 0.05; **Figure 1G**). In colon mucosa, AT-pretreated mice had 11 more abundant taxa and 13 less abundant taxa, BC-pretreated mice had five more abundant taxa and one less abundant taxa, and CON mice had three more abundant taxa compared with controls that did not undergo FMT (*p* < 0.05; **Figure 1H**). These data demonstrate that FMT can induce xenomicrobiota colonization in both jejunum and colon mucosa to reprogram the intestinal microbiota and that AT pretreatment facilitated colonization in the colon.

### Perturbations of Intestinal Microbiota after Antibiotic Usage

**FIGURE 2:**
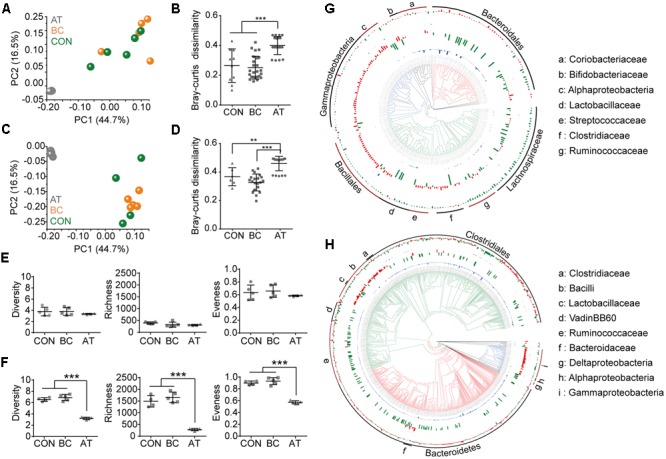
Influence of antibiotic and bowel cleansing treatments on the intestinal mucosa bacterial community. **(A–D)** The mucosa bacterial community in the jejunum **(A,B)** and colon **(C,D)** were analyzed using OTU taxa; the principal coordinate analyses plotted on the first two principal components **(A,C)** and Bray–Curtis dissimilarities **(B,D)** are shown. **(E,F)** Alpha diversity of the mucosa bacterial community in the jejunum **(E)** and colon **(F)**. **(G,H)** Taxonomic distribution of intestinal microbiota in the jejunum **(G)** and colon **(H)** mucus after AT or BC treatment. Taxa with a mean abundance of >10^-4^ are shown. The internal tree presents the taxonomy at the phylum level: Bacteroidetes (red), Proteobacteria (blue), Firmicutes (green), and others (black). The four circular histograms indicate the taxa that are less abundant compared with control mice; from inside to outside, the first and third circular histogram present the taxa depleted by AT or BC treatment, respectively, and the second and fourth circular histogram denote the fold-change of taxa by AT or BC treatment, respectively. The taxa that are more or less abundant after treatment are highlighted in red and blue, respectively. The main bacterial taxa in the intestine are annotated in the outer circle. Data are presented as means ± SD. ^∗∗^*p* < 0.01, ^∗∗∗^*p* < 0.001.

To investigate possible mechanisms of reprogramming efficiency alteration, we assessed the influence of AT or BC treatments on the microbiota in the intestinal mucosa and in the intestinal contents. The mucosa microbiota from different treatments grouped into two clusters in both the jejunum (**Figure 2A**) and colon (**Figure 2C**). Based on Bray–Curtis dissimilarity, AT-treated mice were significantly different from CON mice and from BC-treated mice (*p* < 0.05), but the intestinal microbiota were similar between CON mice and BC-treated mice (**Figure 2B,D**). Multiple indexes (diversity, richness, and evenness) were used to evaluate the microbiota change. Although different treatments did not alter the jejunum microbiota (**Figure 2E**), AT treatment lead to significantly less alpha diversity in the colon microbiota (*p* < 0.05; **Figure 2F**). To verify these findings, the lumen content microbiota from each of the three treatments was also analyzed, and similar results were observed (**Supplementary Figure S4**).

To examine the intestinal mucosa microbiota composition difference after BC or AT treatment, we analyzed 233 and 844 high-confidence OTUs (abundance >10^-4^) in the jejunum and colon, respectively. The mucosa microbiota composition was strongly influenced by AT treatment; after AT treatment, 68 OTUs disappeared, 62 OTUs decreased, and 103 OTUs increased in the jejunum. The increased OTUs mostly corresponded to bacteria belonging to class Alphaproteobacteria or Gammaproteobacteria, order Bacillales, and family Bifidobacteriaceae, Streptococcaceae, or Clostridiaceae (**Figure 2G**). In the colon, after AT treatment, 575 OTUs disappeared, 133 OTUs decreased, and 123 OTUs increased. The increased OTUs mostly belonged to bacteria of class Bacilli, Alphaproteobacteria, or Gammaproteobacteria, and family Clostridiaceae or Bacteroidaceae (**Figure 2H**). At the genus level, 276 taxa and 247 taxa were found in the jejunum and colon mucosa, respectively. Of these, 40 taxa in the jejunum and 50 taxa in the colon significantly increased (*p* < 0.05; **Supplementary Figures S5a,b**). Critically, some of these increased taxa (jejunum: 14 taxa, colon: 19 taxa) were bacteria that are resistant to one or more antibiotics based on the Antibiotic Resistance Genes Database (ARDB) (**Supplementary Figures S5c,d**). These findings confirm that antibiotic usage, even for a short time, disrupts gut microbiota and skews the intestinal microbiota by eliminating the vast antibiotic-sensitive bacteria and reserving the antibiotic-resistant bacteria. This deficiency of indigenous bacteria may leave more niche vacancies available for xenomicrobiota colonization; however, the enrichment in antibiotic-resistant bacteria may pose a risk for animal health.

### Metabolism Change of Intestinal Mucosa Microbiota after Antibiotic Usage

Previous work has demonstrated that antibiotic usage leads to a metabolism change of the intestinal microbiota (Maurice et al., 2013); however, the mucosa microbiota is distinct from the lumen microbiota, and the existence of a similar alteration in the mucosa microbiota metabolism has not been determined. To assess if there is an antibiotic-induced mucosa microbiota metabolism change, we performed a PICRUSt analysis that predicted the microbiota functional profiles and identified 41 second-level and 328 third-level classification KEGG pathways in the jejunum and colon. Among these pathways, 137 and 161 metabolism pathways that were changed after antibiotic usage were identified by LEfSe in the jejunum and colon, respectively (*p* < 0.05; **Supplementary Figures S6a,b**). In both the second-level and third-level pathways, xenobiotic biodegradation and metabolism pathways were significantly increased in the jejunum and colon (*p* < 0.05; **Supplementary Figures S6a,b**). These results verify the antibiotic selection on the intestinal microbiota that was observed via metabolism profile.

### Dynamic Changes in Intestinal Microbiota Following Combination Therapy with FMT and Antibiotics

**FIGURE 3:**
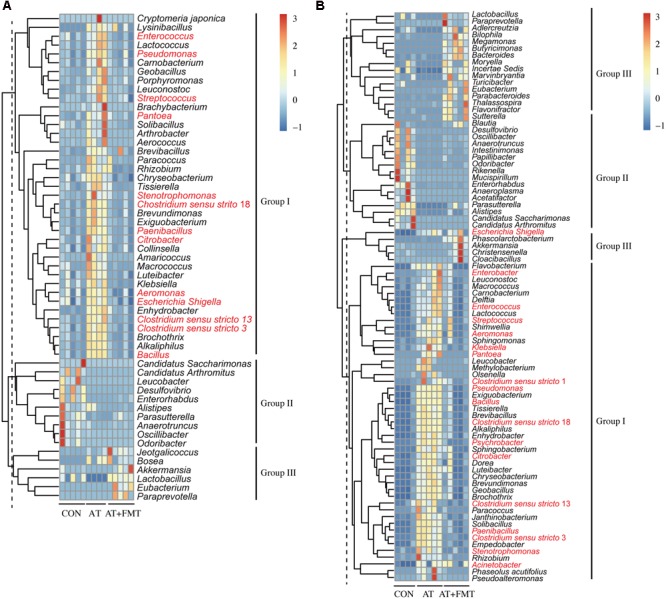
Genus level dynamic changes in the mouse intestinal microbiota after antibiotic pretreatment and FMT. **(A,B)** Changed taxa in the jejunum **(A)** and colon **(B)** are indicated and clustered into three groups. Hierarchical clustering is based on the Euclidean distance. Taxonomic names of the taxa are listed, and names written in red belong to antibiotic-resistant bacteria according to the Antibiotic Resistance Genes Database (ARDB: http://ardb.cbcb.umd.edu/). Only taxa having a change with *p* < 0.05 are shown.

To illustrate the dynamic changes in intestinal microbiota following combination therapy with FMT and antibiotics, the microbiota of control, AT-treated, and AT-pretreated FMT mice were compared at the genus level. In the jejunum and colon, 55 and 84 taxa, respectively, were significantly altered by combination therapy (*p* < 0.05), and these changed bacterial taxa can be grouped into three clusters in either the jejunum or colon (**Figure 3**). Compared with controls, after FMT most of the bacterial taxa (jejunum: 39 taxa, colon: 47 taxa) were recovered (cluster 1), 10 and 16 bacterial taxa in the jejunum and colon, respectively, were un-restorable (cluster 2), 6 and 21 bacterial taxa in the jejunum and colon, respectively, were newly colonized taxa (cluster 3). Although most taxa were restored, the changed taxa may contribute to the overall community change. Our data also demonstrate that amount of antibiotic-resistant bacteria increased after antibiotics usage, but this increase was subsequently eliminated by FMT administration (**Figure 3**).

## Discussion

FMT is an effective and rapid way of reshaping the gut microbiota (Manichanh et al., 2010; Fuentes et al., 2014). Previous work investigated the therapeutic effects of gut preparation with antibiotics or a Moviprep solution prior to performing FMT (Manges et al., 2016), but the efficiency of microbiota alteration caused by these pretreatments has not been tested. Our data demonstrate that FMT can reprogram the intestinal microbiota by introducing xenomicrobiota, but the efficiency is adjustable, and by preparing the gut with antibiotics, FMT efficiency can be elevated in the colon more than it can by preparing the gut with BC or in the absence of a pretreatment.

**FIGURE 4:**
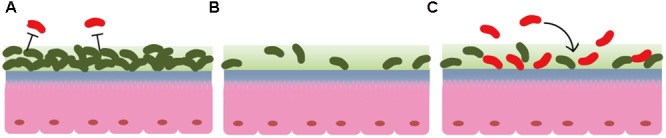
Diagram of the proposed mechanism for antibiotic pretreatment enhancement of FMT efficiency by promoting xenomicrobiota colonization in the intestinal mucosa. **(A)** Commensal bacteria resist xenomicrobiota colonization under normal conditions. **(B)** Antibiotic treatment induces commensal bacteria disruption, and these vacancies provide more available spaces for xenomicrobiota in the mucosa. **(C)** Xenomicrobiota colonization is promoted after antibiotic usage. Symbols in olive indicate commensal bacteria, and those in red indicate xenobacteria.

We also observed that AT treatment induced intestinal microbiota disruption by eliminating vast bacterial taxa. In contrast with observations in a previous report that intestinal microbiota changed immediately after BC and rebounded to its original profile after 14 days (Jalanka et al., 2015), this study found that the intestinal microbiota was not changed after 24 h of BC treatment. Our findings suggest that, prior to FMT administration, the intestinal mucosa in mice treated with BC or left untreated were adhered by indigenous bacteria, unlike the intestinal mucosa in mice treated with AT. Thus, we propose that the available niches in mucosa contribute to the improved FMT reprogramming efficiency following antibiotic pretreatment. The indigenous bacteria resist xenomicrobiota invasion; however, antibiotic treatment induces intestinal microbiota disruption by eliminating vast bacteria taxa, which generates more available niches to be colonized by xenomicrobiota in the intestinal mucosa (**Figure 4**). Theoretically, increasing niche vacancy prior to FMT is an important mechanism by which to improve FMT efficiency, and additional studies are needed to investigate the viability of other procedures of efficiently generating available niches.

Although FMT induced microbiota changes in both the jejunum and colon, the FMT efficiency in the jejunum was less efficient than that in the colon. Previous studies demonstrated that the microbiota in the small intestine is less stable than that in the large intestine (Gu et al., 2013; Kelly et al., 2017; Li et al., 2017), which suggests that the microbiota in the small intestine may be susceptible to environmental factors. In support of this idea, FMT has been demonstrated to be most effective in diseases of the large intestine diseases, such as CDI or IBD (Fuentes et al., 2014; Bagdasarian et al., 2015; Marchesi et al., 2016; Ishikawa et al., 2017). The FMT efficiency on diseases of the small intestine still needs further research.

It is well known that antibiotic usage may induce lesions by disturbing gut microbiota (Sekirov et al., 2008; Becattini et al., 2016; Knoop et al., 2016) and enriching antibiotic-resistant bacteria (Jernberg et al., 2010; Baym et al., 2015). Our data support the previous findings, and additionally illustrate that xenobiotic biodegradation and metabolism pathways of mucosa microbiota increased after antibiotic usage. Our data demonstrate here that after FMT administration, the enriched antibiotic-resistant bacteria taxa decreased in abundance to the original profile, which revealed another potential application for FMT: to drive out the universal gut antibiotic-resistant bacteria.

## Conclusion

Our data demonstrate that FMT efficiency is adjustable by changing how the gut is prepared and that antibiotic pretreatment enhances gut microbiota reprogramming by promoting xenomicrobiota colonization. Additionally, the enriched level of antibiotic-resistant bacteria in the gut that followed antibiotic usage was subsequently eliminated by FMT, which implies that FMT may be an alternative way to drive out universal gut antibiotic-resistant bacteria. These new findings may be instructive for FMT administration, as well as for future research to further improve the gut microbiota reprogramming efficiency.

## Author Contributions

YW, ZC, and SL designed the study; SJ, HY, and TJ performed experiments; HY, CG, JL, and SD collected and prepared samples for sequencing; HY performed sequencing and sequencing analysis with technical assistance from YW; SJ and KY performed statistical interpretation and analyses; SJ and HY took primary responsibility for writing the manuscript. All authors discussed the results and commented on the manuscript.

## Conflict of Interest Statement

The authors declare that the research was conducted in the absence of any commercial or financial relationships that could be construed as a potential conflict of interest.

## Footnotes

1 http://gsa.big.ac.cn

2 https://huttenhower.sph.harvard.edu/galaxy

## Supplementary Material

FIGURE S1 Experimental design. SPF mice (*n* = 4–5 mice per group) received antibiotics (AT), bowel cleansing (BC), or no treatment as a control (CON). After 3 days of treatment, half of the mice from each treatment group were sampled. The remaining mice then received FMT for 3 days, and samples were harvested from these mice at 1 week post-FMT.

FIGURE S2 Intestinal microbiota difference between the FMT donor and mice. **(a–e)** The relative abundance of intestinal microbiota in the donor (*n* = 1) and in different intestinal parts of control mice (*n* = 4–5) are presented at the phylum **(a)**, class **(b)**, order **(c)**, family **(d)**, and genus **(e)** levels.

FIGURE S3 Intestinal mucosa bacterial community alteration after FMT. **(a,b)** High confidence bacterial distribution after FMT in the jejunum **(a)** and colon **(b)** at the genus level. Each row represents a sample from an individual mouse or donor. The taxa with an abundance of >10^-4^ in each sample are highlighted in black.

FIGURE S4 Influence of antibiotic and bowel cleansing treatments on the intestinal bacteria community in the intestinal lumen content. **(a–d)** The lumen content bacterial community in the jejunum **(a,b)** and colon **(c,d)** were analyzed using OTU taxa, and the principal coordinates analyses plotted on the first two principal components **(a,c)** and Bray–Curtis dissimilarities **(b,d)** are shown. **(e,f)** Alpha diversity of the lumen content bacterial community in the jejunum **(e)** and colon **(f)**. Data are presented as means ± SD. ^∗∗^*p* < 0.01, ^∗∗∗^*p* < 0.001.

FIGURE S5 Intestinal microbiota change after antibiotic usage at the genus level. **(a,b)** Heat-map of significantly altered taxa in the jejunum **(a)** and colon **(b)** mucosa. Only taxa with *p* < 0.05 are shown. **(c,d)** Antibiotic-resistant bacteria in the jejunum **(c)** and colon **(d)** mucosa. The ARDB database (http://ardb.cbcb.umd.edu/) was searched for vancomycin-, neomycin-, or ampicillin-resistant bacteria at the genus level. *Clostridium* represents *clostridium sensu stricto 1, clostridium sensu stricto 3, clostridium sensu stricto 13*, and *clostridium sensu stricto 18*. The antibiotic-resistant bacteria in the taxa that were more abundant after antibiotic usage are listed in Venn diagrams.

FIGURE S6 Intestinal microbiota metabolism change after antibiotic usage. **(a,b)** LEfSe cladograms demonstrating the metabolism change of mucosa microbiota in the jejunum **(a)** and colon **(b)**. Each dot represents a metabolism pathway; dots and branches in red or green indicate that metabolism activity is higher or lower after antibiotic usage. Metabolism pathways with *p* < 0.05 and LDA score >2 are presented, and the LDA scores are shown with histograms.
