# Supplementary figures and images for: Preparing the Gut with Antibiotics Enhances Gut Microbiota Reprogramming Efficiency by Promoting Xenomicrobiota Colonization
Source: Front Microbiol. 2017 Jun 28;8:1208. doi: 10.3389/fmicb.2017.01208 (PMC5487471; doi:10.3389/fmicb.2017.01208)

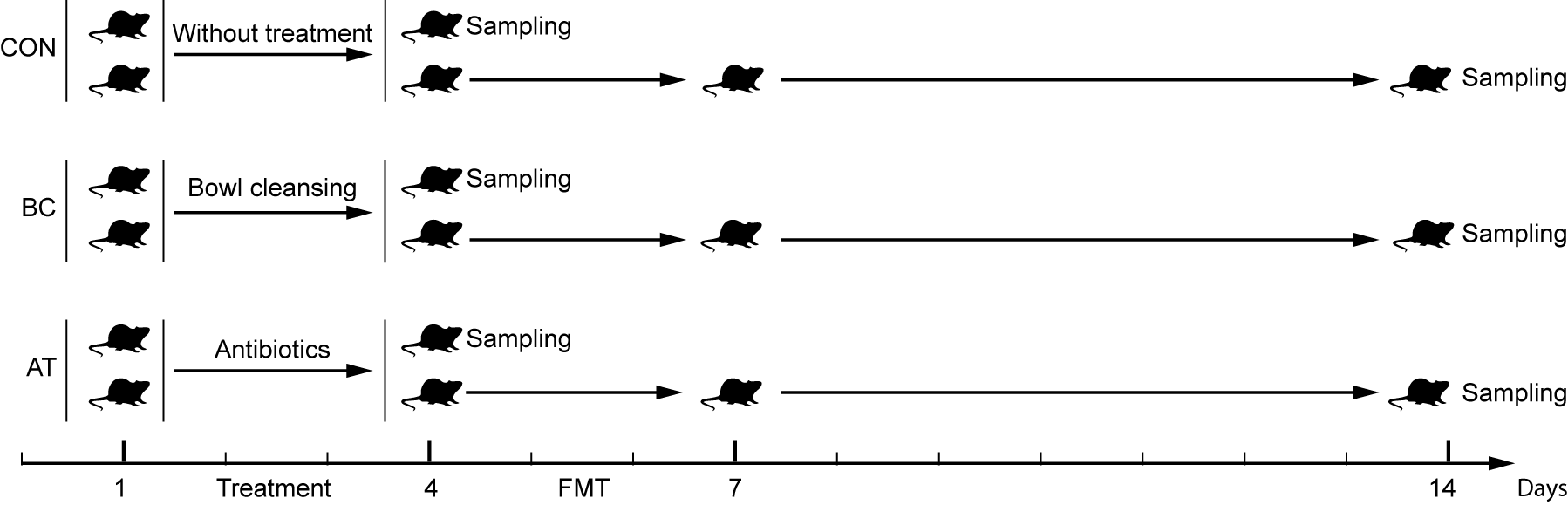

Supplement: FIGURE S1 — Experimental design. SPF mice (n = 4–5 mice per group) received antibiotics (AT), bowel cleansing (BC), or no treatment as a control (CON). After 3 days of treatment, half of the mice from each treatment group were sampled. The remaining mice then received FMT for 3 days, and samples were harvested from these mice at 1 week post-FMT. [file Image_1.tif]

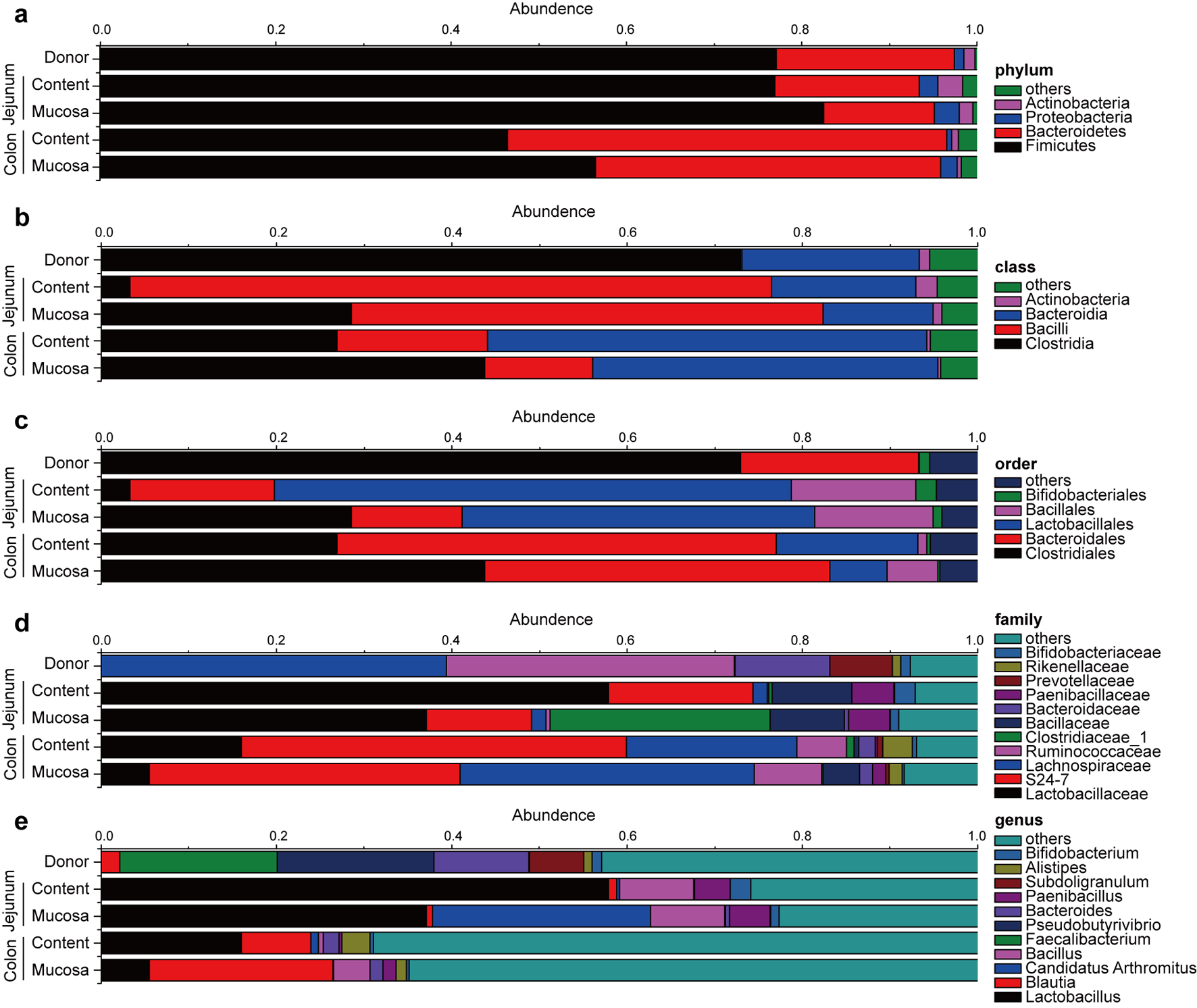

Supplement: FIGURE S2 — Intestinal microbiota difference between the FMT donor and mice. (a–e) The relative abundance of intestinal microbiota in the donor (n = 1) and in different intestinal parts of control mice (n = 4–5) are presented at the phylum (a), class (b), order (c), family (d), and genus (e) levels. [file Image_2.tif]

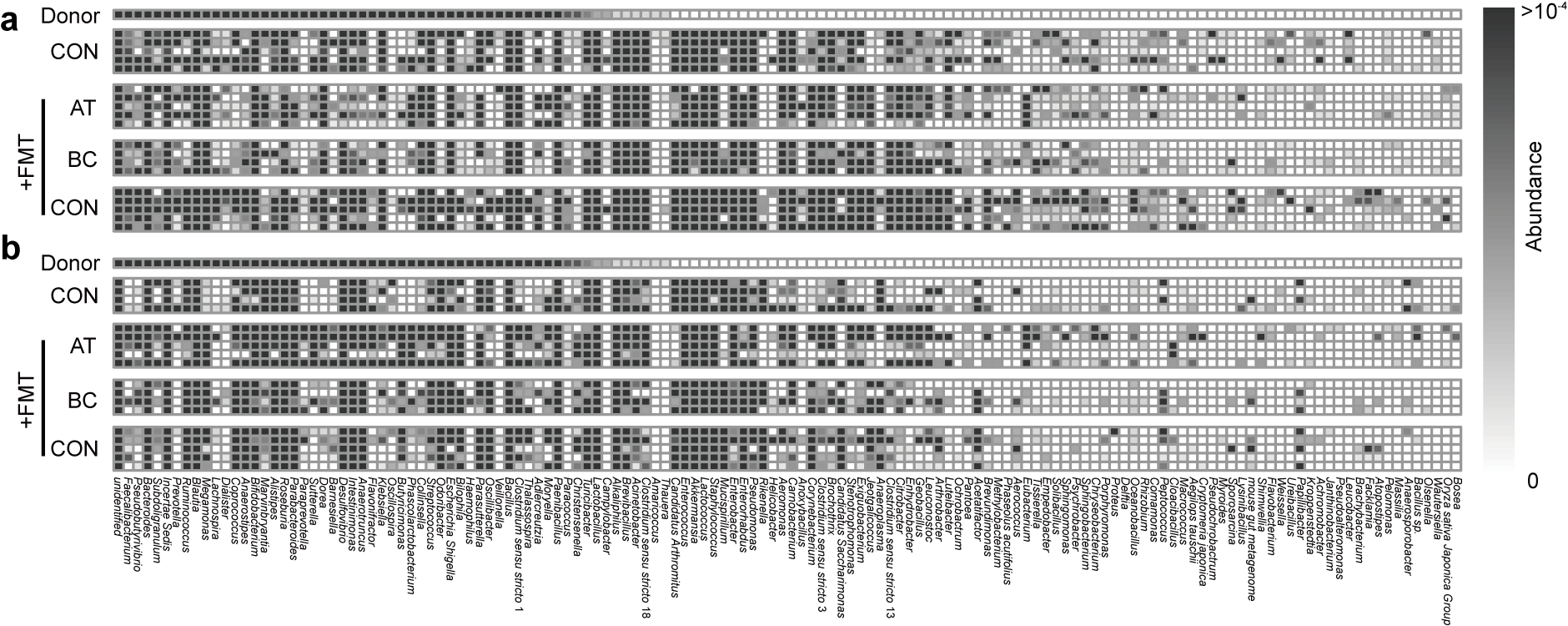

Supplement: FIGURE S3 — Intestinal mucosa bacterial community alteration after FMT. (a,b) High confidence bacterial distribution after FMT in the jejunum (a) and colon (b) at the genus level. Each row represents a sample from an individual mouse or donor. The taxa with an abundance of >10-4 in each sample are highlighted in black. [file Image_3.tif]

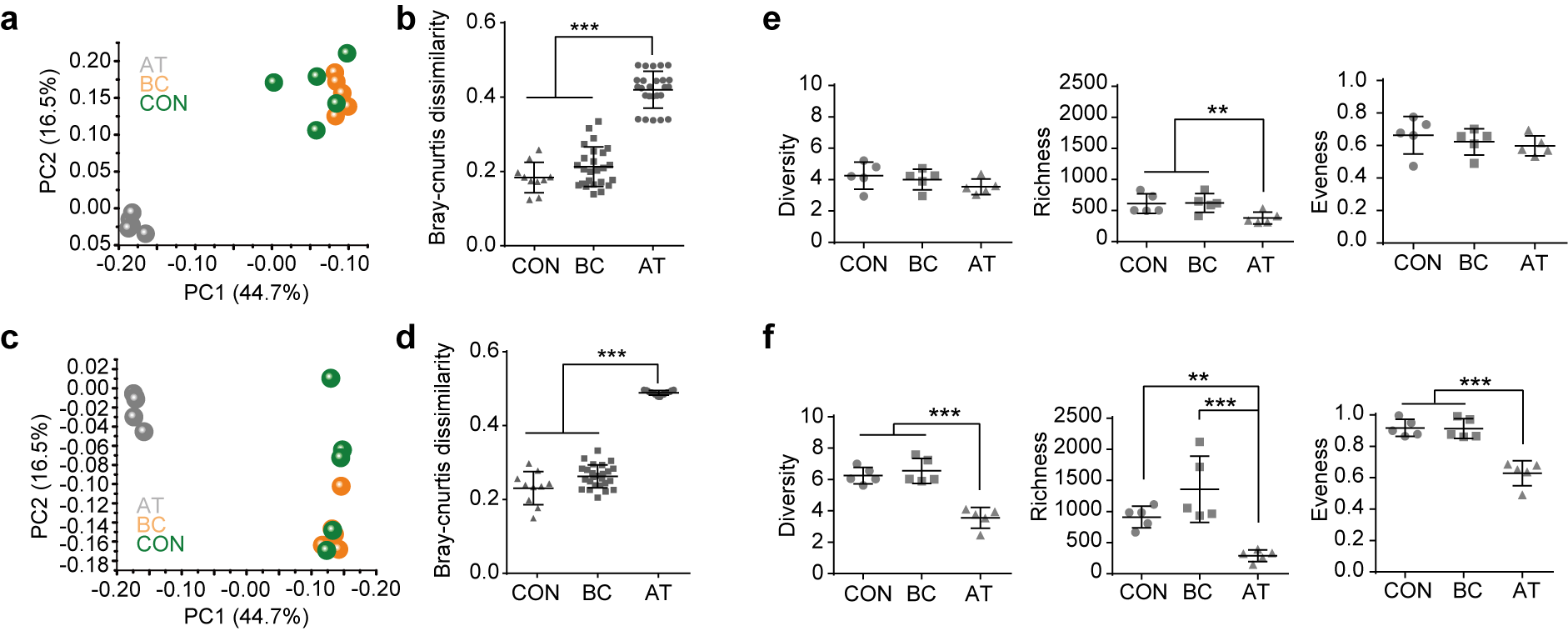

Supplement: FIGURE S4 — Influence of antibiotic and bowel cleansing treatments on the intestinal bacteria community in the intestinal lumen content. (a–d) The lumen content bacterial community in the jejunum (a,b) and colon (c,d) were analyzed using OTU taxa, and the principal coordinates analyses plotted on the first two principal components (a,c) and Bray–Curtis dissimilarities (b,d) are shown. (e,f) Alpha diversity of the lumen content bacterial community in the jejunum (e) and colon (f). Data are presented as means ± SD. ∗∗p < 0.01, ∗∗∗p < 0.001. [file Image_4.tif]

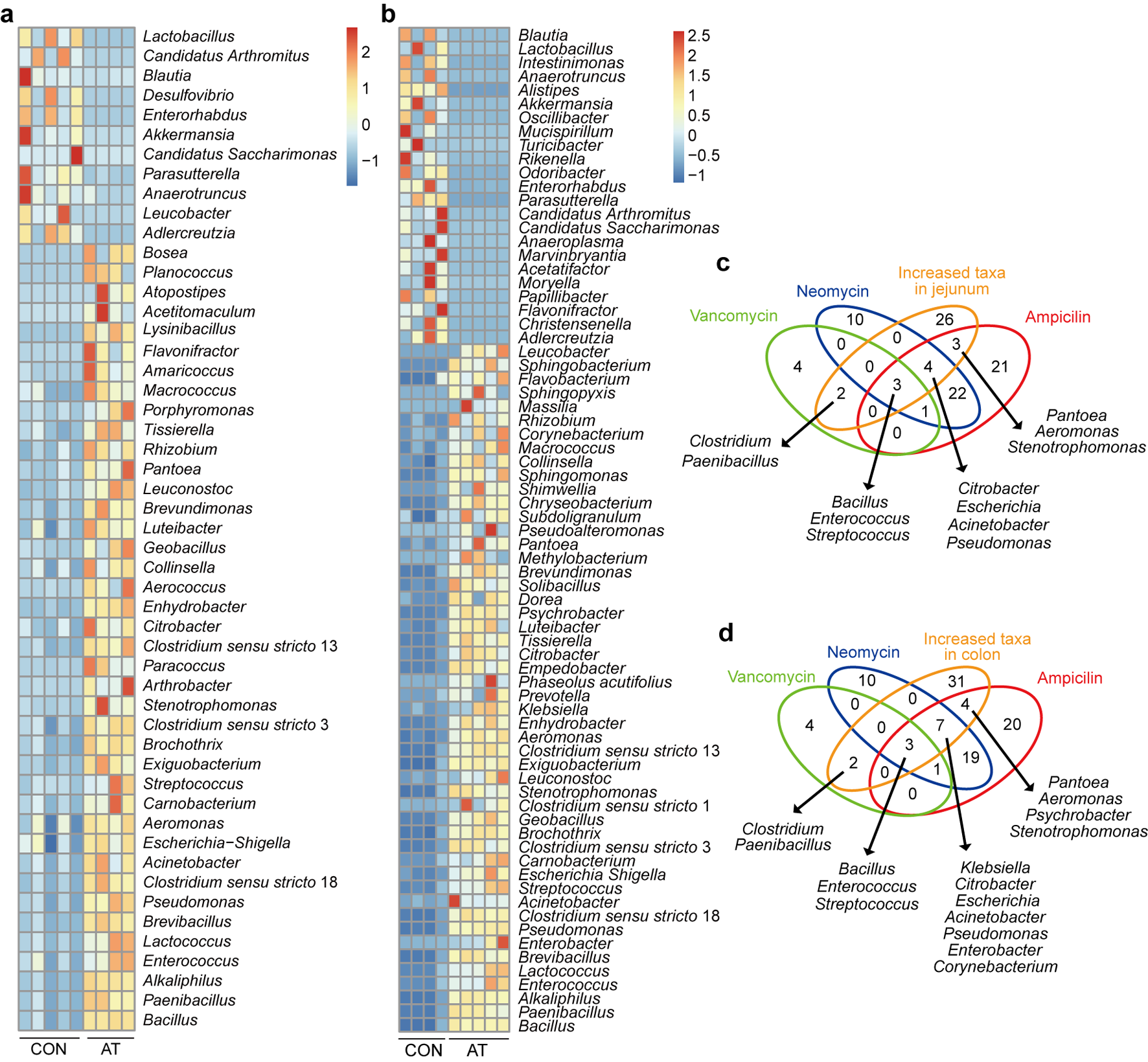

Supplement: FIGURE S5 — Intestinal microbiota change after antibiotic usage at the genus level. (a,b) Heat-map of significantly altered taxa in the jejunum (a) and colon (b) mucosa. Only taxa with p < 0.05 are shown. (c,d) Antibiotic-resistant bacteria in the jejunum (c) and colon (d) mucosa. The ARDB database (http://ardb.cbcb.umd.edu/) was searched for vancomycin-, neomycin-, or ampicillin-resistant bacteria at the genus level. Clostridium represents clostridium sensu stricto 1, clostridium sensu stricto 3, clostridium sensu stricto 13, and clostridium sensu stricto 18. The antibiotic-resistant bacteria in the taxa that were more abundant after antibiotic usage are listed in Venn diagrams. [file Image_5.tif]

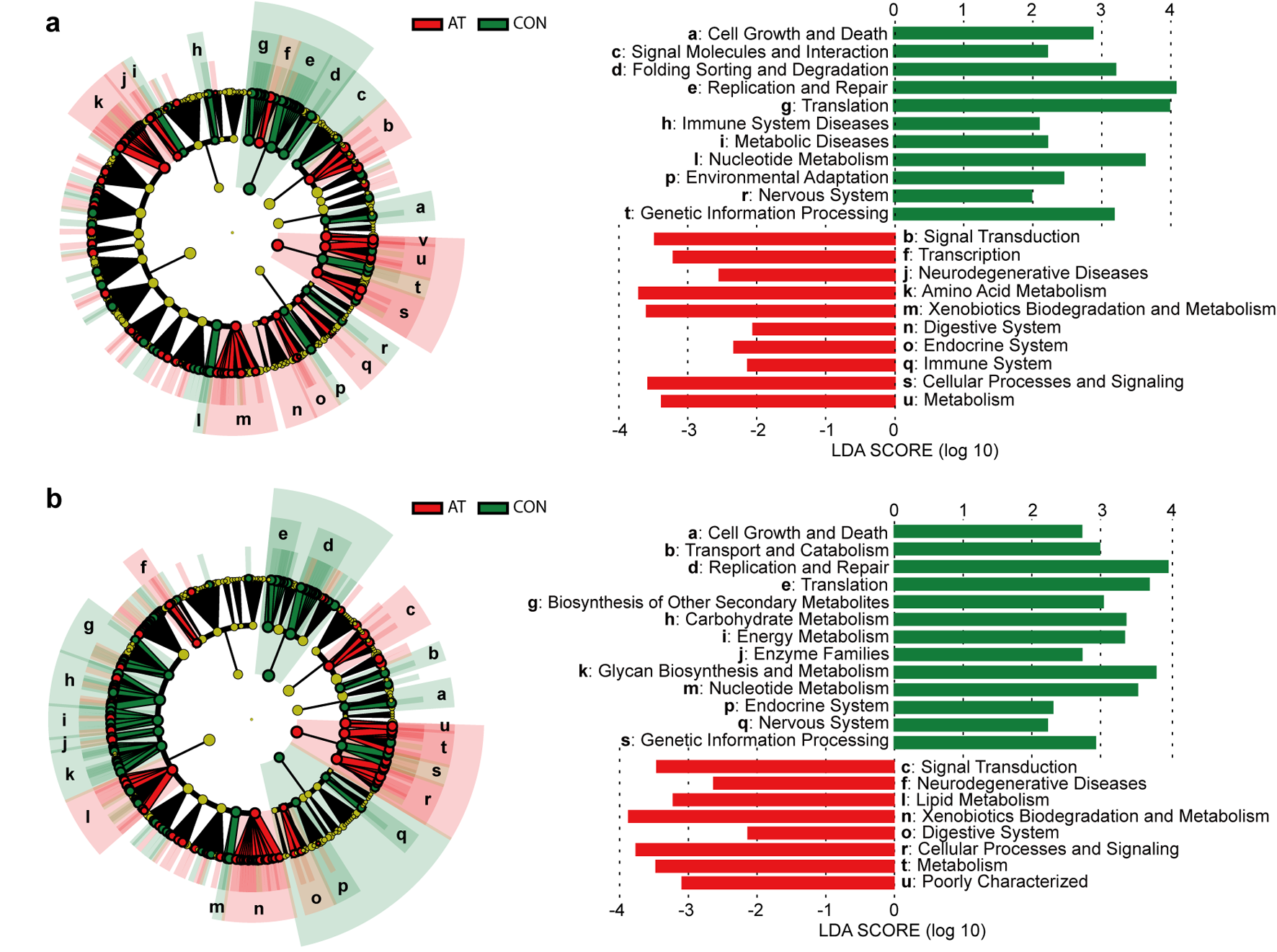

Supplement: FIGURE S6 — Intestinal microbiota metabolism change after antibiotic usage. (a,b) LEfSe cladograms demonstrating the metabolism change of mucosa microbiota in the jejunum (a) and colon (b). Each dot represents a metabolism pathway; dots and branches in red or green indicate that metabolism activity is higher or lower after antibiotic usage. Metabolism pathways with p < 0.05 and LDA score >2 are presented, and the LDA scores are shown with histograms. [file Image_6.tif]
